# Prediction of influenza-like illness based on the improved artificial tree algorithm and artificial neural network

**DOI:** 10.1038/s41598-018-23075-1

**Published:** 2018-03-20

**Authors:** Hongping Hu, Haiyan Wang, Feng Wang, Daniel Langley, Adrian Avram, Maoxing Liu

**Affiliations:** 1 School of Science, North University of China, Taiyuan, Shanxi 030051 PR China; 2 School of Mathematical and Natural Sciences, Arizona State University, Phoenix, Arizona USA

## Abstract

Because influenza is a contagious respiratory illness that seriously threatens public health, accurate real-time prediction of influenza outbreaks may help save lives. In this paper, we use the Twitter data set and the United States Centers for Disease Control’s influenza-like illness (ILI) data set to predict a nearly real-time regional unweighted percentage ILI in the United States by use of an artificial neural network optimized by the improved artificial tree algorithm. The results show that the proposed method is an efficient approach to real-time prediction.

## Introduction

Influenza can lead to serious illness, and influenza-like illnesses (ILI) can and do cause death. Therefore, it is crucial to public health that accurate real-time monitoring, early detection, and prediction of influenza outbreaks are provided. Disease detection and surveillance systems provide epidemiologic intelligence that help health officials to draw up preventive measures and assist clinic and hospital administrators in making optimal staffing and stocking decisions^1^.

ILI is defined by the World Health Organization (WHO) as an acute respiratory infection with a measured fever higher than 38 °C, and cough, with onset within the previous 10 days^2^. In a February 2016 document for outpatient illness surveillance, ILI is defined by the US Centers for Disease Control and Prevention (CDC) defined ILI as “ever (temperature of 100°*F*[37.8 °C] or greater) and a cough and/or a sore throat without a known cause other than influenza^3^”.

Research has revealed that elevated risk of ILI is associated with factors such as active or passive smoking^4–8^. For example, Wang *et al*.^8^ determine an association between passive smoking and ILI risk among housewives in North China, and have observed the effects of gene polymorphism related to the metabolism of smoking pollutants. Additionally, researchers are focusing on accurate real-time monitoring, early detection and prediction of influenza outbreaks such as using machine learning to predict the percentage ILI (%ILI)^9^.

From the web site https://gis.cdc.gov/grasp/fluview/fluportaldashboard.html for 10 regions defined by Health and Human Services (HHS), we can see the weighted %ILI, the unweighted %ILI, the numbers of patients age 0–4, age 5–24, age 25–64 and age 65, ILI total and total patients. According to Santillana *et al*.^9^, the CDC’s ILI data provides useful estimates of influenza activity with a known time lag of one to two weeks. This time lag has an influence on public health decisions. Thus many attempts have been made to provide real-time estimates of ILI in the US in an indirect manner^10–17^. Google Flu Trends (GFT) used Internet searches to predict ILI in the US, making it the most widely used nontraditional prediction method in the past few years^18^. But GFT was shut down in August 2015. This cessation left a need for novel and reliable methods to fill the gap. Santillana *et al*.^9^ proposed a real-time monitoring model for ILI, which they call ARES (“AutoRegressive Electronic health Record support vector machine”) to predict the CDC’s ILI for all geographic US regions including the nation and ten regions defined by HHS for the three flu seasons spanning 2012 to 2015. The results showed that ARES solved the prediction problem when compared with dynamic linear regression and a two-term autoregressive model.

Many methods for predictions and classifications exist. Among them, there are machine learning^9^ for ILI, the artificial neural network^19^ for air quality index (AQI), PDE^20^ for prediction-error expansion-based reversible data hiding, finite element modeling^21^ for prediction of muscle activation for an eye movement, and a time-space discretization approach^22^ for bus travel time prediction.

Here, we focus on the neural network for prediction. The BP neural network (BPNN)^23,24^, the self-organization map neural network^25^, the radial basis function (RBF) neural network^19^, the wavelet neural network^26,27^, and the generalized radial basis function (GRBF) neural network^28^ are used to perform predictions and classifications. The randomness of the artificial networks’ initial parameters generalizes the predictions & classifications. Therefore, there are population-based algorithms proposed to optimize these initial parameters. For example, in Qiu and Song^24^, a genetic algorithm was used to optimize the initial parameters of a BP neural network for Japanese stock forecasting. In Lu *et al.*^19^ and in Lu *et al*.^28^, particle swarm optimization algorithm was used to optimize the initial parameters of RBF for predicting AQI and GRBF neural networks for predicting the Chinese stock index, respectively.

A novel population-based algorithm, the artificial tree (AT) algorithm^29^, was proposed in 2017; it simulated tree growth and photosynthesis. In this paper, the AT algorithm is improved to optimize the initial parameters of BP neural network for predicting the unweighted %ILI by use of the CDC data set and the Twitter data set. We name our model IAT-BPNN, which stands for *improved AT optimizing BP neural network*.

## Methods

### The artificial tree algorithm

Inspired by the growth law of trees, in 2017 Li *et al*.^29^ proposed a kind of population-based algorithm, the artificial tree (AT) algorithm, to perform thirty typical benchmark problems.

AT is similar to the common geometric features of the trees. AT algorithm is the optimization process of the problems, which is similar to the transfer process of the organic matter produced by the photosynthesis in the leaves from the leaves to the tree trunk through adjacent twigs and then through the thick branches. For the optimization problem, every solution is a *D*-dimension vector, which stands for the branch of AT and whose component denotes the branch position. Here, the *i*^*th*^ branch position is denoted as ${x}_{i}=({x}_{i1},{x}_{i2},\,\cdots ,\,{x}_{iD})$, $(i=\mathrm{1,}\,\mathrm{2,}\,\cdots ,\,SN)$, where *SN* is the number of branches and *D* is the number of the variables in the optimized problem. In AT algorithm, a better solution denotes a thicker branch and the best solution represents the tree trunk.

#### Generate the initial branches

The initial branches population is generated randomly by Eq. (1).

1 $${x}_{ij}={x}_{ij}^{min}+{rand}\mathrm{(0,}\,\mathrm{1)}\times ({x}_{ij}^{max}-{x}_{ij}^{min}),\,(i=\mathrm{1,}\,\mathrm{2,}\,\cdots ,SN,j=\mathrm{1,}\,\mathrm{2,}\,\cdots ,D),$$

where ${x}_{ij}^{max}$ and ${x}_{ij}^{min}$ are the upper and lower boundaries for the *j*^*th*^ variable of the *i*^*th*^ branch, respectively, and *rand*(0, 1) is a random number between 0 and 1. For these branches, the corresponding solutions are calculated and then the optimal solution and the corresponding branch are regarded as the best solution *f*(*x*_*best*_) and the best branch *x*_*best*_.

#### Branch territory

According to the transfer of organic matter, it is key for AT algorithm to update the branch in some way. In AT, there are three branch update methods: crossover behavior, self-evolution behavior and random behavior. These updated theories depend on the branch territory. In AT algorithm, every branch owns its territory. And the total number of branches fall into a certain range within one territory. The territory of a thicker branch is obtained from Eq. (2).

2 $${V}_{i}({x}_{i})=(L+L\times fit({x}_{i}))\times 2.$$

where *L* is a constant, *V*_*i*_(*x*_*i*_) is the branch territory, and *fit*(*x*_*i*_) is the fitness value of the branch *x*_*i*_. The larger *fit*(*x*_*i*_) is, the better is the branch *x*_*i*_. For the minimum problem, the *fit*(*x*_*i*_) is calculated as follows:

3 $${fit}({x}_{i})=\{\begin{array}{ll}1/(f({x}_{i})+\mathrm{1)} & if\,f({x}_{i})\ge \mathrm{0,}\\ 0 & if\,f({x}_{i}) < 0\end{array}$$

where *f*(*x*_*i*_) is solution of the branch *x*_*i*_.

The Euclidean distance between the *i*^*th*^ branch *x*_*i*_ and the *j*^*th*^ branch *x*_*j*_ is denoted by Eq. (4).

4 $${Di}{{s}}_{ij}={norm}({x}_{i}-{x}_{j}\mathrm{)}.$$

The crowded tolerance *Tol* is proposed on the basis of the Euclidean distance. The territory of the branch *x*_*i*_ can be expressed as *Dis*_*ij*_ < *V*_*i*_(*x*_*i*_). *Nb* denotes the number of other branches within this territory. The relation of *Nb* and *Tol* is to determine whether the branch territory is crowded.

#### Self-evolution operator and crossover operator

For the branch *x*_*i*_, if *Nb* > *Tol*, it is crowded in the territory of *x*_*i*_. Thus the self-evolution is carried out to renew the branch as follows:

5 $${x}_{new}={x}_{i}+{rand}\mathrm{(0,}\,\mathrm{1)}\times ({x}_{best}-{x}_{i}),$$

Otherwise, the crossover operator is performed to obtain the evolution of the branch. The new branch *x*_*new*_ is merged with a randomly generated branch

6 $${x}_{0}={x}_{i}+{rand}(-\mathrm{1,}\,\mathrm{1)}\times ({V}_{i}({x}_{i})/\mathrm{2)}$$

within half of the branch territory and the current branch *x*_*i*_ by stochastic linear interpolation as follows:

7 $${x}_{new}={rand}\mathrm{(0,}\,\mathrm{1)}\times {x}_{0}+{rand}\mathrm{(0,}\,\mathrm{1)}\times {x}_{i},$$

where *rand*(−1, 1) is a random number between −1 and 1.

#### Random operator

If the new branch generated by the crossover operator or the self-evolution operator is thicker than the old branch, the new branch replaces the old one. Otherwise, this new branch is abandoned and another new branch is generated by the crossover operator or the self-evolution operator. When the search number reaches the maximum search number *Li*(*x*_*i*_) = *N* × *fit*(*x*_*i*_) + *N* which is proportional to the fitness value *fit*(*x*_*i*_) and there is no new branch superior to the original one, where *N* is a constant, no better branch within this territory exists. Therefore, the original operator is replaced by the random operator and a new branch is randomly generated.

#### Update the optimal value

The solutions of each branch are compared with each other and the thickest branch in the round of cycle is obtained. For the minimum problem, $f({x}_{i})(i=\mathrm{1,}\,\mathrm{2,}\,\cdots ,\,SN)$ is the solution of the branch *x*_*i*_ and $f({x}_{0}^{best})=\,{\min }(f({x}_{1}),\,f({x}_{2}),\,\cdots ,\,f({x}_{SN}))$ is recorded as the best solution in the current cycle where the corresponding branch ${x}_{0}^{best}$ is the best branch. The best solution is chosen from the previous and current solutions. If the best solution of the previous cycle is better, the solution and branch are replaced by the previous best ones. Otherwise, keep the current best solution.

### The Improved Artificial Tree Algorithm

In artificial tree algorithm, a self-evolution operator is improved by means of the probability *p*. If *p* > 0.5, self-evolution operator is carried out by use of Eq. (5). Otherwise, let *max*(*x*_*i*_) denote the maximum component of branch *x*_*i*_ and *s* denote the position of *max*(*x*_*i*_) in *x*_*i*_. If *max*(*x*_*i*_) is positive, the *s*^*th*^ component of *x*_*i*_ is replaced by 1 − *max*(*x*_*i*_); otherwise, the *s*^*th*^ component of *x*_*i*_ is replaced by 1 + *max*(*x*_*i*_). Thus the artificial tree algorithm is improved, abbreviated as IAT.

## Experiments

### Data

In this paper, we select two kinds of data sets for research on ILI prediction: the CDC data set and the Twitter data set. These two kinds consist of 55 weeks of data between the 41^*st*^ week in 2016 and the 45^*th*^ week in 2017 and are extracted according to the partition from CDC defined by HHS in USA.

#### The CDC data set

The CDC is a unit of the US Department of HHS, which provides reliable information for the protection of public health and safety, and makes healthy decision to improve citizens’ health through partnerships between the national health department and other organizations. The CDC data are regularly tracking reported visits to doctors according to the CDC official statistics on the trends of influenza or outbreaks such as SARS and Ebola. In the United States, the CDC records the number of people seeking medical attention with ILI symptoms. The agency’s web site https://gis.cdc.gov/grasp/fluview/fluportaldashboard.html provides both new and historical data, where CDC’s ILI is freely distributed and available through ILInet^30^. From this web site, we can obtain the CDC’s data set on unweighted %ILI.

#### The Twitter data set

Twitter is a website of social network service and microblogging service based on US, and allows users to update messages up to 140 characters in length. Twitter can be used to track users’ casual remarks about their feelings when they would give them self-diagnosis and could suffer from allergies, strep infections, or common colds as well as real cases of influenza. Wang *et al*.^31^ have built a prototype of flu-surveillance system and developed a dynamic spatial-temporal PDE model that can predict flu prevalence in both spatial and temporal dimensions at both national and regional levels. It designs, implements, and evaluates a prototype system that automatically collects, analyzes and models geo-tagged flu tweets from real-time Twitter data streams. Specifically, flu tweets are extracted from real-time data streams and each tweet is tagged with geographical locations based on three information sources: (i) the geographical location in the profile of the user who tweeted the message, (ii) the physical location where the user sent the tweet and enabled their geographical location tracking in the Twitter App, and (iii) the geographical location mentioned in the content of the tweets. The Twitter data for this paper are collected from the system we built in Wang *et al*.^31^.

To evaluate the algorithm for prediction, mean squared error (MSE)^19^, relative mean squared error (RMSE)^19^, and mean absolute percentage error (MAPE)^19^ are taken as the criteria standards, whose formulae are as follows:

8 $${MSE}=\frac{1}{n}\sum _{i\mathrm{=1}}^{n}{({y}_{i}-{x}_{i})}^{2},$$

9 $${RMSE}=\frac{1}{n}\sum _{i\mathrm{=1}}^{n}{(\frac{{y}_{i}-{x}_{i}}{{y}_{i}})}^{2},$$

10 $${MAPE}=\frac{1}{n}\sum _{i\mathrm{=1}}^{n}|\frac{{y}_{i}-{x}_{i}}{{y}_{i}}|\times 100.$$

where *y*_*i*_ denotes the *i*^*th*^ actual value, and *x*_*i*_ denotes the *i*^*th*^ predicted value.

### Analysis

We set up a set of regional models for predicting the the unweighted percentage ILI (%ILI) in the United States. In these models, the independent variables used to predict the real-time estimates of ILI activity at week *t* include the (*t* − 3)^*th*^ week, the (*t* − 2)^*th*^ week and the (*t* − 1)^*th*^ week of the unweighted percentage ILI (%ILI) in the CDC’s data set, and the (*t* − 1)^*th*^ week of twitter data in the Twitter data set.

In this paper, AT algorithm and IAT algorithm are used to optimize the parameters of BP neural networks for prediction of %ILI, respectively, thus optimized models are obtained and written as AT-BPNN and IAT-BPNN, respectively. For comparison purposed throughout the paper, we produced real-time estimates using three models: the basic BPNN, AT-BPNN and IAT-BPNN. Therefore the inputs of all three models are composed of the (*t* − 3)^*th*^ week, the (*t* − 2)^*th*^ week and the (*t* − 1)^*th*^ week of %ILI in the CDC’s data set and the (*t* − 1)^*th*^ week of twitter data in the Twitter data set, and the outputs of all three models are the *t*^*th*^ week of % ILI in the CDC’s data set. Thus 52 4-dimension samples are obtained, where 47 samples are taken to be trained and 5 samples are taken to be tested. The number of the neural nodes in the only hidden layer of BPNN part in every model is taken as 8 and then the structure of BPNN part is 4-8-1. Then, BPNN, AT-BPNN and IAT-BPNN are performed for the above samples to predict the %ILI.

## Results

**Figure 1:**
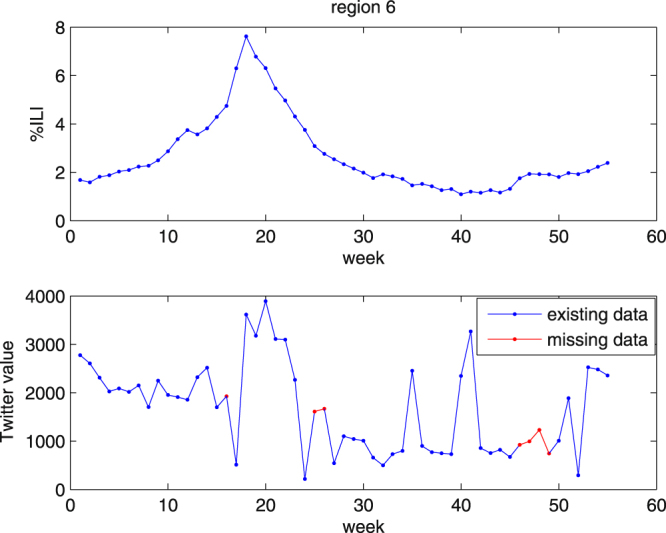
% ILI and the twitter data of region 6.

First, we use the basic BP neural network for prediction to revise some missing data. We perform ten times and take the corresponding prediction of the missing data with the minimum MAPE. For example, The Twitter data for region 6 in Fig. 1 misses the 16th; 25th–26th; 46th–49th data. The red dots in Fig. 1 represent our predictions.

**Figure 2:**
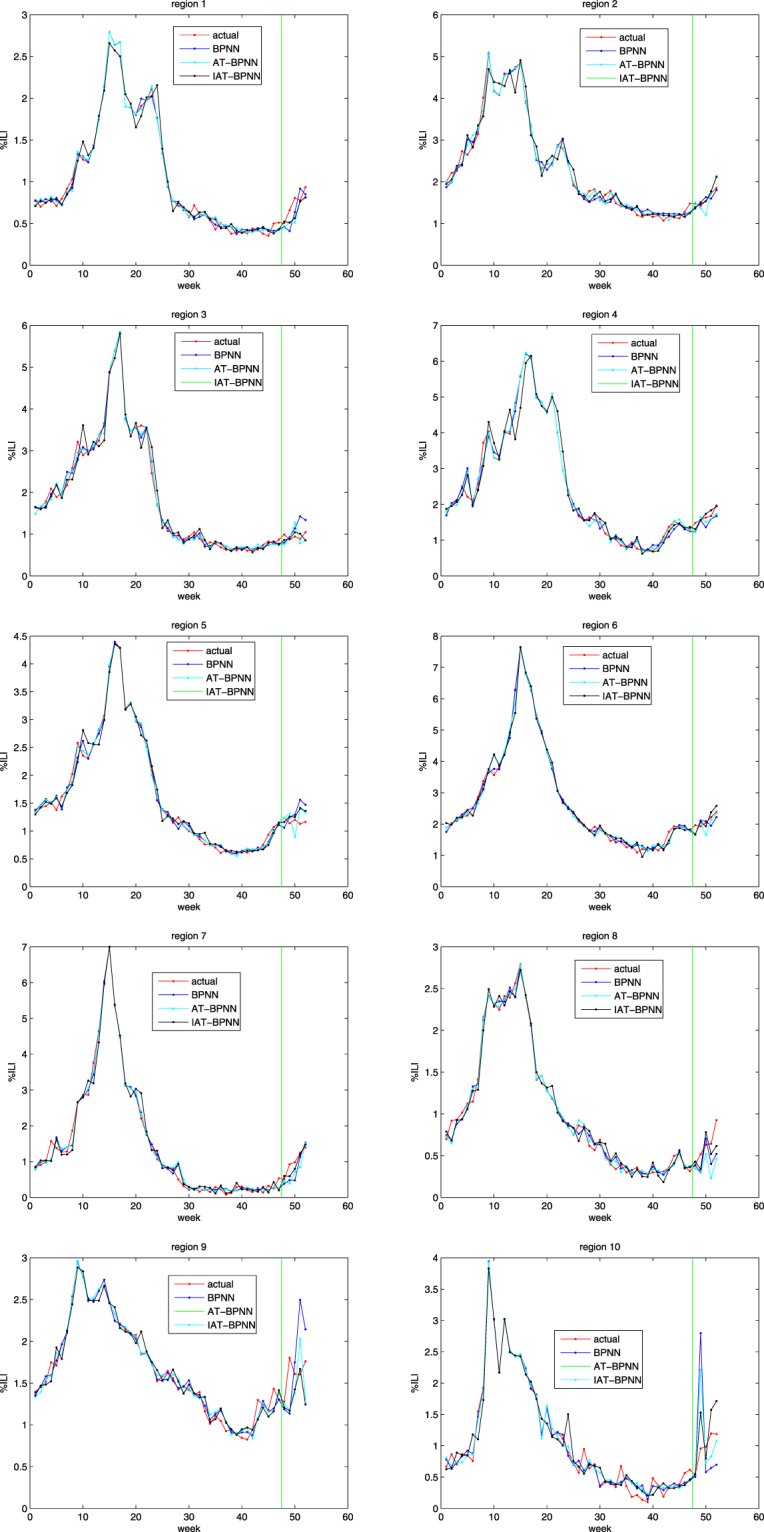
The trained and tested results of 10 regions.

To predict the %ILI by use of the above 52 samples, we perform three models: BPNN, AT-BPNN and IAT-BPNN. In BPNN and the BPNN part in AT-BPNN and IAT-BPNN, the training maximum iterations is 10,000, the learning rate is 0.002, the momentum factor is 0.95, and the training goal is 0.00001. In addition, the size of population is 60; the AT algorithm and the IAT algorithm are all run 500 times. The structures of BPNN and the BPNN part in AT-BPNN and IAT-BPNN are all 4-8-1, where 4, 8 and 1 denote the numbers of the nodes in the input layer, in the hidden layer and in the output layer, respectively. Forty-seven samples are trained and five samples are tested. From the experiments, we obtain Table 1 and Fig. 2.

**Table 1:** MSE, RMSE, and MAPE of three models for 10 regions of USA.

region	error	BPNN	AT-BPNN	IAT-BPNN
1	mse	0.0241	0.0254	0.0191
	rmse	0.0475	0.0422	0.0320
	mape	0.1898	0.1688	0.1427
2	mse	0.0100	0.0402	0.0163
	rmse	0.0041	0.0149	0.0051
	mape	0.0613	0.0893	0.0526
3	mse	0.0919	0.0420	0.0164
	rmse	0.1075	0.0447	0.0170
	mape	0.2729	0.1824	0.1186
4	mse	0.0466	0.0280	0.0134
	rmse	0.0165	0.0102	0.0054
	mape	0.1099	0.0912	0.0594
5	mse	0.0673	0.0454	0.0284
	rmse	0.0509	0.0336	0.0217
	mape	0.1941	0.1586	0.1256
6	mse	0.0448	0.0494	0.0374
	rmse	0.0103	0.0121	0.0088
	mape	0.0921	0.0888	0.0872
7	mse	0.1007	0.1088	0.0322
	rmse	0.1155	0.1084	0.0399
	mape	0.2879	0.2796	0.1631
8	mse	0.0549	0.0904	0.0333
	rmse	0.1043	0.1790	0.0668
	mape	0.2744	0.3723	0.2404
9	mse	0.2831	0.1653	0.1414
	rmse	0.1016	0.0554	0.0453
	mape	0.2610	0.2060	0.1714
10	mse	0.8188	0.3583	0.1569
	rmse	0.8519	0.3794	0.1387
	mape	0.6532	0.3973	0.3134

Figure 2 shows the actual value and predicted values of three models on the trained samples and the tested samples of ten regions, where the green line perpendicular to the horizontal axis in every subfigure divides the whole plate into two parts: the left part is the actual outputs and the predicted outputs of the trained data on three models and the right part is the actual outputs and the predicted outputs of the tested data on three models. From Fig. 2, we can see that the outputs of these three models are close to the actual output in the trained state and there are differences between the predicted outputs of the three models and the actual outputs in the tested state.

Table 1 shows the MSE, RMSE, and MAPE on the tested samples of ten regions. The increasing orders of these three models on the MSE are IAT-BPNN, BPNN and AT-BPNN on region 1 and region 6-region 8, are BPNN, IAT-BPNN and AT-BPNN only on region 2, and are IAI-BPNN, AT-BPNN and BPNN on region 3-region 5 and region 9-region 10. The increasing orders of these three models on the RMSE are IAT-BPNN, AT-BPNN and BPNN on region 1, region 3-region 5, region 7 and region 9-region 10, are BPNN, IAT-BPNN and AT-BPNN only on region 2, and are IAT-BPNN, BPNN and AT-BPNN on region 6 and region 8. The increasing orders of these three models on the MAPE are IAT-BPNN, AT-BPNN and BPNN on region 1, region 3-region 5, region 7 and region 9-region 10, and are IAI-BPNN, BPNN and AT-BPNN on region 2, region 6 and region 8. Therefore, according to these three errors, the proposed model, IAT-BPNN, is suitable for the prediction of influenza-like illness.

And also from Table 1, the average MSEs of BPNN, AT-BPNN and IAT-BPNN across all ten regions in the tested period are 0.1542, 0.0953, and 0.0495, respectively; the average RMSEs of BPNN, AT-BPNN and IAT-BPNN across all ten regions in the tested period are 0.1410, 0.0880, and 0.0381, respectively; the average MAPEs of BPNN, AT-BPNN and IAT-BPNN across all ten regions in the tested period are 0.2397, 0.2034, and 0.1474, respectively. From Table 1, we also find the errors of three models on region 10 are the biggest. Therefore, the proposed model, IAT-BPNN, is superior to AT-BPNN and BPNN for predicting CDC’s %ILI as defined by HHS.

## Discussion

In this study, the Twitter data and the CDC’s data containing 55 weeks’ data between the 41^*st*^ week in 2016 and the 45^*th*^ week in 2017, in combination with an improved population-based artificial tree algorithm optimizing the parameters of BP neural network are capable of accurately predicting real-time influenza activity at the regional scales in the US.

The ability of CDC’s data and Twitter data to predict CDC’s ILI regionally was established using three dynamically-trained models: BPNN, AT-BPNN and IAT-BPNN. The results show that incorporating CDC’s ILI and the Twitter’s influenza data, using a suitable improved artificial tree optimizing the parameters of BP neural network, can improve influenza predictions.

Table 1 shows that using IAT-BPNN reduced errors (MSE, RMSE, and MAPE) when compared to BPNN and AT-BPNN. MSE across regions was generally improved, with the largest improvement in region 10 (from 0.8188 to 0.1569) and the mildest reduction taking place in region 1 (from 0.0254 to 0.0191). The average MSE generally improved, with the greatest performance in region 10 and the mildest reduction in region 6. RMSE across regions was generally improved, with the largest improvement in region 10 (from 0.8519 to 0.1387) and the mildest reduction taking place in region 6 (from 0.0121 to 0.0088). The average RMSE generally improved, with the greatest performance in region 10 and the mildest reduction in region 7. MAPE across regions was generally improved, with the largest improvement in region 10 (from 0.6532 to 0.3134) and the mildest reduction taking place in region 6 (from 0.0921 to 0.0872). The average MAPE generally improved, with the greatest performance in region 10 and the mildest reduction in region 1.

The only region on MSE and RMSE where the combination of historical CDC data and the Twitter data did not lead to improvements when compared to the BPNN was region 2, where MSE went from 0.0100 to 0.0163 and RMSE went from 0.0041 to 0.0051. For 9 out of the 10 regions, IAT-BPNN correctly estimated the real-time CDC’s %ILI.

In this study, the Twitter data have been revised by use of the basic BP neural network. And we would like to note that we used the Twitter data and the CDC data to train all of our models dynamically. BPNN and the BP parts of AT-BPNN and IAT-BPNN have set the same parameters, and AT and IAT have the same setting. Our experience training near real-time influenza prediction models has shown us that the results of IAT-BPNN are in contrast to those of BPNN and AT-BPNN. There are many discrepancies between the influenza estimates using IAT-BPNN and the actual CDC values, as captured by MSE, RMSE and MAPE, which are comparable to those using BPNN and AT-BPNN. The experimental results showed that IAT-BPNN outperforms BPNN and AT-BPNN. We hope that future work will use IAT-BPNN for predicting ILI at the state and city levels, in other countries as well as for other communicable diseases. Differently improved artificial tree algorithms will be proposed to optimize the parameters of artificial neural networks for many applications.

## Conclusion

In this paper, we proposed an improved artificial tree (IAT) to optimize the parameters of BP neural network(IAT-BPNN) for predicting the CDC’s %ILI of USA. The inputs consist of the %ILI data derived from CDC of USA and Twitter data. Compared with AT-BPNN and BPNN, IAT-BPNN is fit for solving this problem. The prediction of IAT-BPNN for %ILI are not only suitable for ten regions defined by HHS, but it also provides that the population algorithms can be applied and improved to optimize the parameters of artificial neural networks for solving the predictive problem. From Fig. 2 and Table 1, we also find that differences between the actual values and the predicted values exist. These may exist for four main reasons: revised Twitter data, generalization of the artificial neural network, the structure of BPNN and the part of BP neural network in AT-BPNN and IAT-BPNN and one year’s time series. Continuing work is needed to improve the current algorithms or to propose the new algorithm to optimize the parameters of artificial neural networks for diminishing the generalization.
